# Reprogrammable Phase‐Transition Composites for Adaptive Dynamic Shape Morphing

**DOI:** 10.1002/advs.202523219

**Published:** 2026-01-21

**Authors:** Yiding Zhong, Wei Tang, Xinyu Guo, Kecheng Qin, Pingan Zhu, Qincheng Sheng, Yonghao Wang, Huayong Yang, Jun Zou

**Affiliations:** ^1^ State Key Laboratory of Fluid Power and Mechatronic Systems School of Mechanical Engineering Zhejiang University Hangzhou China

**Keywords:** adaptive soft robotics, energy storage, phase‐transition composites, reprogrammable deformation, shape locking

## Abstract

Adaptive dynamic deformation has attracted growing attention because of its great significance for robots to adapt to the environment. However, designing a flexible smart material with reprogrammable and local programmable regulation for adaptive dynamic deformation in robotic systems is a substantial challenge. In nature, phase transitions are used to shape biological tissues, modulate growth shape through stiffness changes and provide growth momentum through fluid pressure. Inspired by this, we report a reprogrammable phase‐transition composites that uses the stiffness change induced by reversible solid‐liquid phase transition to program and regulate the material deformation actuated by reversible liquid‐vapor phase transition, thereby achieving adaptive dynamic deformation in a controllable manner. By regulating the order of the two phase transitions, phase‐transition composites can achieve not only reprogrammable deformation and local programmable deformation, but also rapid deformation and shape locking. We have developed a series of functional enhancements and applications using phase‐transition composites, demonstrating the effectiveness of the composite phase‐transition programmed deformation modulation mechanism. This mechanism enables robots to achieve reversible active deformation modulation and reprogrammable deformation modulation, opening a door for robotic systems with adaptive dynamic deformation.

## Graphical abstract

This paper reports a bio‐inspired reprogrammable phase‐transition composites that uses the stiffness change induced by reversible solid‐liquid phase transition to program and regulate the material deformation actuated by reversible liquid‐vapor phase transition, thereby achieving adaptive dynamic deformation in a controllable manner, and enabling reprogrammable deformation, local programmable deformation, rapid deformation and shape locking.

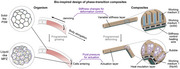

## 1 Introduction

Adaptive dynamic deformation is of great significance for robots to adapt to complex environment [1, 2, 3]. By adjusting the morphology and corresponding motion modes, robots can enhance their capabilities, including adapting to different environments, tasks or expanding functions. For example, through deformation, robots can change their motion modes to move in different media [4, 5], can cross obstacles or pass through narrow gaps to adapt to unstructured environments [6, 7], and can adaptively grasp or manipulate objects of different shapes and sizes [8, 9].

In order to realize and utilize the adaptive dynamic deformation of the robotic system, it is necessary to have a rich variety of deformation forms and to be able to accurately program and regulate the deformation to achieve on‐demand adaptive deformation. With the development of soft robotics [10, 11, 12, 13] and stretchable electronics [14, 15, 16], many deformable robots that can adjust their shapes to achieve different motion modes have been reported. Researchers have proposed drones that passively deform to adjust motion modes [17], universal grippers that passively adapt [18], etc., but these studies lack reversible active deformation adaptation capabilities and can only be reconfigured by external forces. Some robots use flexible smart materials to achieve active controllable deformation regulation and state switching, including origami deformable wheels [19], clay sculptured robotic skin [20], amphibious turtle robot [5], etc. However, the origami‐based deformable structures can only achieve preset deformations in limited discrete positions, the variable states of the clay sculptured robotic skin are limited to shapes with circular cross‐sections, and the fins of the amphibious turtle robot can only change between two certain states. It can be seen that the deformable robots in existing research have a single deformation form and are difficult to program and regulate. They have few degrees of deformation freedom and can often only switch between a few certain states, and lack the ability to be reprogrammed into rich and adjustable states. Moreover, most existing research can only achieve overall uniform deformation and cannot achieve local programmable deformation only at the desired deformation site. Therefore, there is currently a lack of a flexible smart material that can achieve reprogrammable and local programmable regulation for adaptive dynamic deformation of robotic systems.

Most soft robots are inspired by natural organisms. In nature, phase transitions often occur in biological systems, generally caused by the interaction between cells and neighboring cells and the extracellular medium, and are used to shape biological tissues [21, 22]. This involves the combination of two effects: one is that the organism regulates the growth process through stiffness changes caused by phase transition behavior; the other is that the organism provides fluid pressure as the actuating force for growth through cell proliferation and migration. The combination of these two effects ultimately results in anisotropic controlled growth or deformation. For example, during the development of the vertebrate body axis, the cell population that leaves the mesodermal progenitor zone (MPZ) and matures into the presomitic mesoderm (PSM) undergoes a jamming transition from fluid‐like behavior to solid‐like behavior. This liquid‐solid phase transition behavior regulates growth through changes in stiffness in specific regions. At the same time, as cells from the dorsal medial area enter the MPZ, the fluid pressure increases, providing the actuating force for growth and deformation. The two effects work together to allow for controlled unidirectional elongation of the body axis [23, 24], and is able to maintain the structure, achieving a function similar to shape locking.

Inspired by the behavior of organisms regulating growth shape through stiffness changes and providing growth power through fluid pressure, we proposed a class of reprogrammable phase‐transition composites that combines the effects of solid‐liquid phase transition and liquid‐vapor phase transition. The solid‐liquid phase transition exhibits a significant stiffness change (more than 1,000 times [25]), while the liquid‐vapor phase change can generate high fluid pressure (more than 200 kPa [26]) and large deformation output. Phase‐transition composites use the stiffness change caused by the reversible solid‐liquid phase transition of the working medium to program and control the material deformation, and use the reversible liquid‐vapor phase transition to provide the actuating fluid pressure, so that adaptive dynamic deformation can be achieved in a controllable manner. By regulating the sequence of solid‐liquid phase transition and liquid‐vapor phase transition, phase‐transition composites can achieve different working modes such as programmed deformation, rapid deformation and shape locking. By virtue of the reversibility of phase transitions, phase‐transition composites can achieve a variety of reprogrammable deformations and local programmable deformations by selectively activating heating wires at different positions, and can also be adaptively deformed according to the target contour. The response speed of the phase transition actuation method is greatly accelerated through energy storage‐release regulation. The deformation caused by the liquid‐vapor phase transition can be locked by the reversible solid‐liquid phase transition, avoiding the continuous energy consumption required for the liquid‐vapor phase transition flexible actuation to maintain the deformation. Based on phase‐transition composites, we designed functional expansions and applications such as reprogrammable morphing lattices, reprogrammable morphing surfaces, reconfigurable antennas, and amphibious robots, which proved its application value and provided a platform for studying the adaptive dynamic deformation of robotic systems.

## 2 Results

### 2.1 Bio‐Inspired Design of Phase‐Transition Composites

**FIGURE 1:**
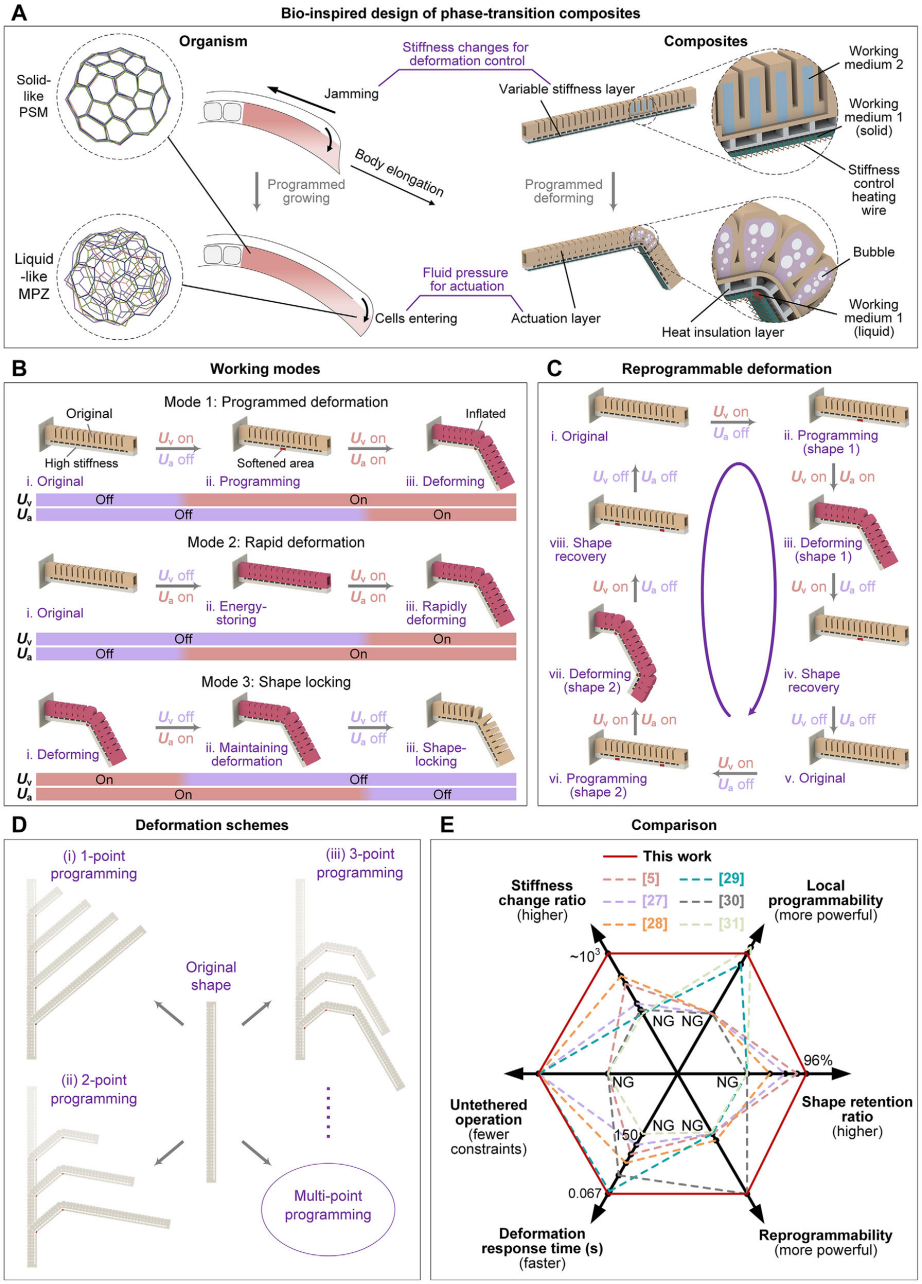
Design and working principle of phase‐transition composites. (A) Bio‐inspired design and deformation modulation principles of phase‐transition composites. Organism: stiffness changes for deformation control—cells jamming and stiffness increasing; fluid pressure for actuation—cells entering and pressure increasing. Phase‐transition composites: stiffness changes for deformation control—variable stiffness layer based on solid‐liquid phase transition; fluid pressure for actuation—actuation layer based on liquid‐vapor phase transition. The transition from liquid‐like mesodermal progenitor zone (MPZ) to solid‐like presomitic mesoderm (PSM) is due to a reduction in both active fluctuations and extracellular spaces. As shown in the enlarged schematic on the left, MPZ has faster dynamics of cell shapes, while PSM has largely static cell boundaries. (B) Schematic diagram of different working modes, including (i) programmed deformation, (ii) rapid deformation, and (iii) shape locking. *U*_v_ refers to the voltage applied to the stiffness control heating wire, and *U*_a_ refers to the voltage applied to the actuation heating wire. The on/off of *U*_v_ is used to control the softening/hardening of the set area of the variable stiffness layer, and the on/off of *U*_a_ is used to control the deformation/recovery of the actuation layer. (C) Schematic diagram of the reprogrammable deformation process. (D) Schematic diagram of different deformation schemes of the same reprogrammable phase‐transition composites, including 1‐point, 2‐point, and 3‐point programming. (E) The distinct advantages of our phase‐transition composites in six aspects compared to other flexible smart materials for deformable structures. NG stands for not given.

In nature, due to the different interaction characteristics of microscopic components such as cells, biological systems exhibit a rich phenomenology of matter states on a macroscopic level, including different fluidity and stiffness, and can transform between these matter states, which is similar to the phase transitions of matter between solid, liquid, and gas states [21, 22]. This biological phase transition plays an important role in the development of organisms. For example, as shown in Figure 1A, during the formation of the anterior‐posterior body axis in animal development, the cell population that leaves the MPZ and forms the PSM undergoes a jamming transition similar to a liquid‐solid phase transition, thereby programming and regulating growth through changes in stiffness in specific areas. At the same time, cells from the dorsal medial area flow into the MPZ, providing fluid pressure as the actuating force for growth and deformation. In this way, under the combined effects of deformation regulation based on phase transition stiffness changes and actuation based on fluid pressure, the body axis is controlled to elongate unidirectionally [23, 24]. As shown in Figure 1A, inspired by this biological deformation regulation strategy, we propose a class of reprogrammable phase‐transition composites that can controllably achieve adaptive dynamic deformation. It uses the solid‐liquid phase transition of working medium 1 to regulate stiffness to achieve deformation programming, and uses the liquid‐vapor phase transition of working medium 2 to provide fluid actuating pressure. The phase‐transition composites mainly include an actuation layer, a variable stiffness layer and a heat insulation layer. The actuation layer contains working medium 2, and the variable stiffness layer contains working medium 1. Both layers are embedded with heating wires, and the heat insulation layer is used to avoid mutual thermal interference between the two layers. The local reversible solid‐liquid phase transition of the working medium 1 in the variable stiffness layer is used to adjust its local stiffness change, while the reversible liquid‐vapor phase transition of the working medium 2 in the actuation layer is used to produce the overall actuating deformation. In this way, the phase‐transition composites can not only achieve deformation only in the set area, but also can be repeatedly reprogrammed into a rich variety of different deformation forms after manufacturing.

As shown in Figure 1B, by adjusting the order of the two types of phase transition, the phase‐transition composites can achieve different modes including programmed deformation, rapid deformation and shape locking. In the programmed deformation mode (Movie S1), the stiffness control heating wire in the local area is first controlled to work so as to heat the variable stiffness layer to soften the set area, and then the actuation layer is heated. The phase‐transition composite material deforms only in the softened area according to the programming of the variable stiffness layer, thereby realizing local programmed control. After the working medium 2 is cooled, the phase‐transition composite material returns to its original shape due to the elasticity of the silicone shell itself, and is re‐fixed in the initial shape after the working medium 1 is cooled and solidified, thereby realizing reversible deformation control. In the rapid deformation mode (Movie S2), the working medium 2 is first heated to cause the expansion of the actuation layer. However, since the variable stiffness layer is in a high stiffness state as a whole, the deformation is limited, and the volume expansion of the actuation layer is converted into elastic potential energy and stored in the phase‐transition composites. Subsequently, the variable stiffness layer is heated, and as the solid‐liquid phase transition of the working medium 1 in the set area occurs, the elastic potential energy stored in the previous stage is suddenly released, and the phase‐transition composites undergo rapid deformation in the local softening area of the variable stiffness layer. In this way, rapid deformation is achieved through controllable energy storage and release, thereby improving the response speed of the phase transition actuation method. In the shape locking mode (Figure S4 and Movie S3), for the phase‐transition composites in a deformed state, the variable stiffness layer is first cooled to allow the working medium 1 in the softened area to cool and solidify, and then the actuation layer is cooled to lock the phase‐transition composites in the desired deformation state. In this way, the shape locking of the phase‐transition composites is achieved, thereby avoiding the continuous energy input required to maintain the deformation and reducing the energy consumption of the phase transition actuation method. The reversibility of the solid‐liquid phase transition of working medium 1 and the reversibility of the liquid‐vapor phase transition of working medium 2 endow the phase‐transition composites with the ability to achieve reprogrammable deformation. As shown in Figure 1C, by utilizing its reversible deformation ability and selectively programming its stiffness, the phase‐transition composites can be repeatedly reprogrammed into rich and adjustable different deformation shapes, thereby improving its adaptability to different application scenarios. With this reprogrammable deformation ability, the phase‐transition composites can be programmed multiple times to achieve a rich variety of deformation types. As shown in Figure 1D, by heating and programming one, two or three points on the variable stiffness layer, the phase‐transition composites can achieve a variety of deformation forms at different deformation positions, proving its rich and adjustable deformation state. As shown in Figure 1E, compared with existing flexible smart materials for deformable structures [5, 27, 28, 29, 30, 31], our phase‐transition composites show obvious advantages in reprogrammability, local programmability, stiffness change ratio, shape retention ratio, deformation response time and untethered operation.

### 2.2 Characterization of Programmed Deformation, Rapid Deformation, and Shape Locking

**FIGURE 2:**
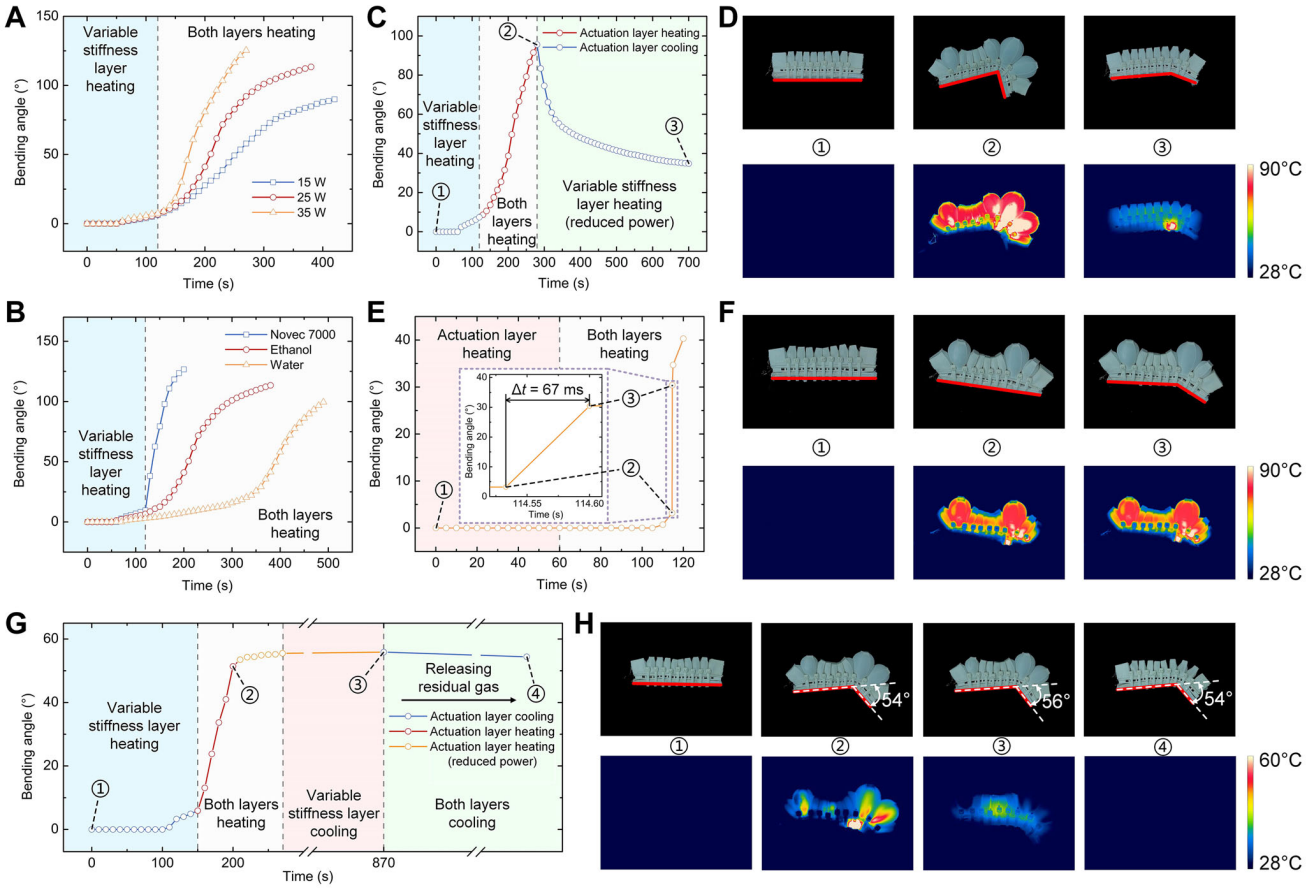
Actuating characteristics of phase‐transition composites under different working modes. (A) Effect of different actuating heating powers on the programmed deformation process of phase‐transition composites. (B) Effect of different boiling points of working medium 2 on the programmed deformation process of phase‐transition composites. (C) Reversible deformation‐recovery process of phase‐transition composites during programmed deformation. (D) Optical and infrared (IR) image sequences corresponding to different states in (C). (E) Time response of phase‐transition composites during rapid deformation process. (F) Optical and IR image sequences corresponding to different states in (E). (G) Time response of phase‐transition composites during shape locking process. (H) Optical and IR image sequences corresponding to different states in (G).

Phase‐transition composites have different actuating characteristics in different working modes. The characterization in the programmed deformation mode adopted a single‐point programmed deformation method. As shown in Figure 2A, the heating power of the actuation layer was an important factor affecting the response speed and the bending angle achieved, and the actuating process can be controlled by adjusting the heating power. As shown in Figure 2B, the type of working medium 2 in the actuation layer also had an important influence on the actuating process. Taking three working media with different boiling points, Novec 7000 (boiling point 34°C), ethanol (boiling point 78°C), and water (boiling point 100°C) as examples, using a working medium 2 with a lower boiling point can obtain a faster actuating speed and achieve a larger bending angle. The reversibility of the liquid‐vapor phase transition ensures the reversible actuation of the phase‐transition composites. As shown in Figure 2C,D, during the deformation‐recovery process, while keeping the programming area of the variable stiffness layer heated and softened, the actuation layer was first heated to undergo programmed deformation, and then the actuation layer was cooled to restore the initial state, and most of the bending deformation can be recovered. As shown in Figure 2E,F, in the rapid deformation mode, the actuation layer was first heated while keeping the variable stiffness layer in a high stiffness state, and the elastic potential energy was stored in the phase‐transition composites at this stage. Then, the programmed position on the variable stiffness layer was heated, and the working medium 1 at the programmed position underwent a solid‐liquid phase change, and the elastic potential energy stored in the previous stage was suddenly released. This also caused the phase‐transition composites to deform rapidly, and the deformation response time was as short as 67 ms, proving its ability to achieve rapid deformation using the method of controllable energy storage and release. As shown in Figure S5, the phase‐transition composites did not show significant performance degradation in 10 repeated cycles under this mode, demonstrating their good repeatability and dynamic stability, comparable to other state‐of‐the‐art research [32]. In addition, after long‐term use, the performance of the phase‐transition composites can be restored by replenishing the working medium 2 in the actuation layer. In the shape locking mode, as shown in Figure 2G,H and Figure S4, after heating the programmed position of the variable stiffness layer and the actuation layer in sequence, the phase‐transition composites underwent programmed deformation. Then, the variable stiffness layer was cooled while maintaining the actuated angle, and the working medium 1 melted in the programmed position was re‐solidified, and the variable stiffness layer recovered the high stiffness state. Subsequently, the actuation layer was cooled, and the phase‐transition composites was locked at the actuated angle. At this time, even if the residual gas in the actuation layer was released, the actuated angle of the phase‐transition composites could still be maintained. In order to characterize the deformation retention capability in the shape locking mode, we define the deformation retention ratio in the shape locking mode as:

(1) $$R_{r}=\frac{\theta_{r}}{\theta_{\max}}$$

where *θ*_r_ is the angle maintained after cooling, and *θ*_max_ is the maximum deformation angle reached before stopping heating the actuating layer. By calculating *R*_r_, we can evaluate the deformation retention effect of the phase‐transition composites. Under the experimental conditions studied (Figure 2G,H), *R*_r_ can reach 96%, proving that the composite phase transition lock strategy can maintain most of the deformation.

### 2.3 Simulation and Adaptive Deformation Modulation

**FIGURE 3:**
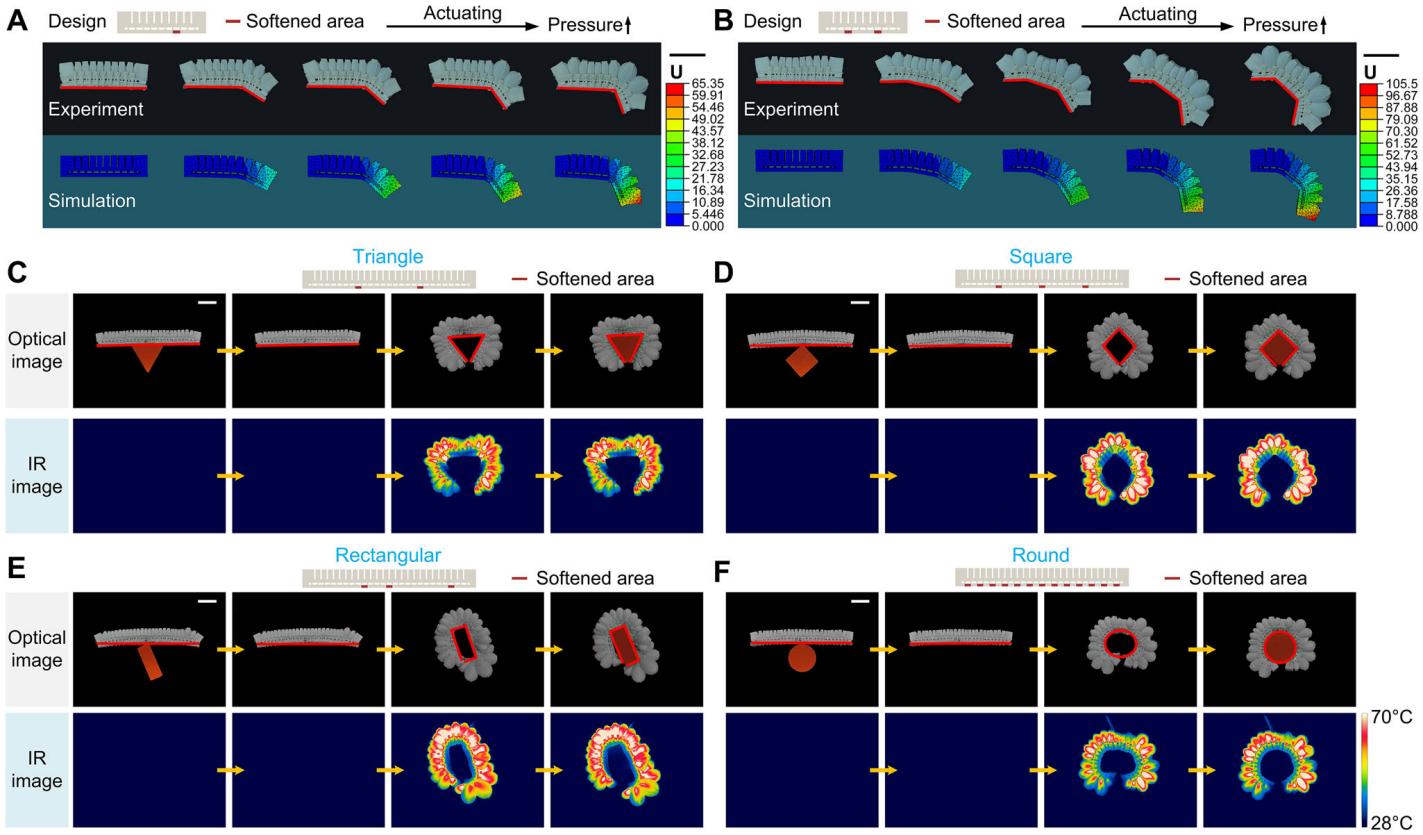
Finite element simulation and adaptive deformation modulation. Experimental verification and finite element simulation of the deformation process of phase‐transition composites under (A) 1‐point programming and (B) 2‐point programming. The process of phase‐transition composites adaptively deforming into (C) triangle, (D) square, (E) rectangle, and (F) round. The red line indicates the controlled deformation contour of the phase‐transition composites. Scale bars, 5 cm.

Finite element simulation was performed here using ABAQUS to predict the deformation of phase‐transition composites, thereby guiding its design process. The fluid pressure provided by the liquid‐vapor phase transition of the working medium 2 was applied to the actuation layer in an equivalent way, that is, in ABAQUS, the pressure was applied as a load to the inner surface of the actuation layer shell. As shown in Figure 3A,B, the deformation of phase‐transition composites under 1‐point programming and 2‐point programming were simulated by finite element simulation and experimentally verified. It can be seen that in both cases, the finite element simulation prediction results were in good agreement with the experimental results, which verified the reliability and accuracy of the finite element simulation model and illustrated the feasibility of using it to guide the design process.

The local programmable deformation ability and reprogrammable deformation ability of phase‐transition composites enable it to be used to achieve adaptive deformation for objects with different shapes, that is, to deform into a shape suitable for the corresponding outer contour according to the shape of different objects. According to the outer contour shape of the object, the stiffness control heating wire at the corresponding position in the variable stiffness layer was activated, and the solid‐liquid phase transition occurred in the working medium 1 at these positions; then the actuation heating wire was activated, and the liquid‐vapor phase transition occurred in the working medium 2; in this way, the phase‐transition composites deformed adaptively. As shown in Figure 3C–F, when 2 or 3 points in the variable stiffness layer were softened, the phase‐transition composites could adapt to triangular, square and rectangular objects in turn, and when the entire variable stiffness layer was softened, the phase‐transition composites could adapt to round object. These demonstrations prove the adaptive dynamic deformation ability of phase‐transition composites and lay the foundation for their use in the development of environmentally adaptive robots. Phase‐transition composites achieve local programmable deformation through local stiffness regulation. This method has a certain scale scalability, but there is a spatial resolution limit when reducing the scale. Because working medium 1 has good thermal conductivity, in order to prevent possible thermal interference between adjacent heating points in the variable stiffness layer, the minimum distance between two adjacent heating points used in the experiments was 0.8 cm. At this scale, the phase‐transition composites can still realize local programmable deformation modulation in experiments.

### 2.4 Reprogrammable Morphing Lattice

**FIGURE 4:**
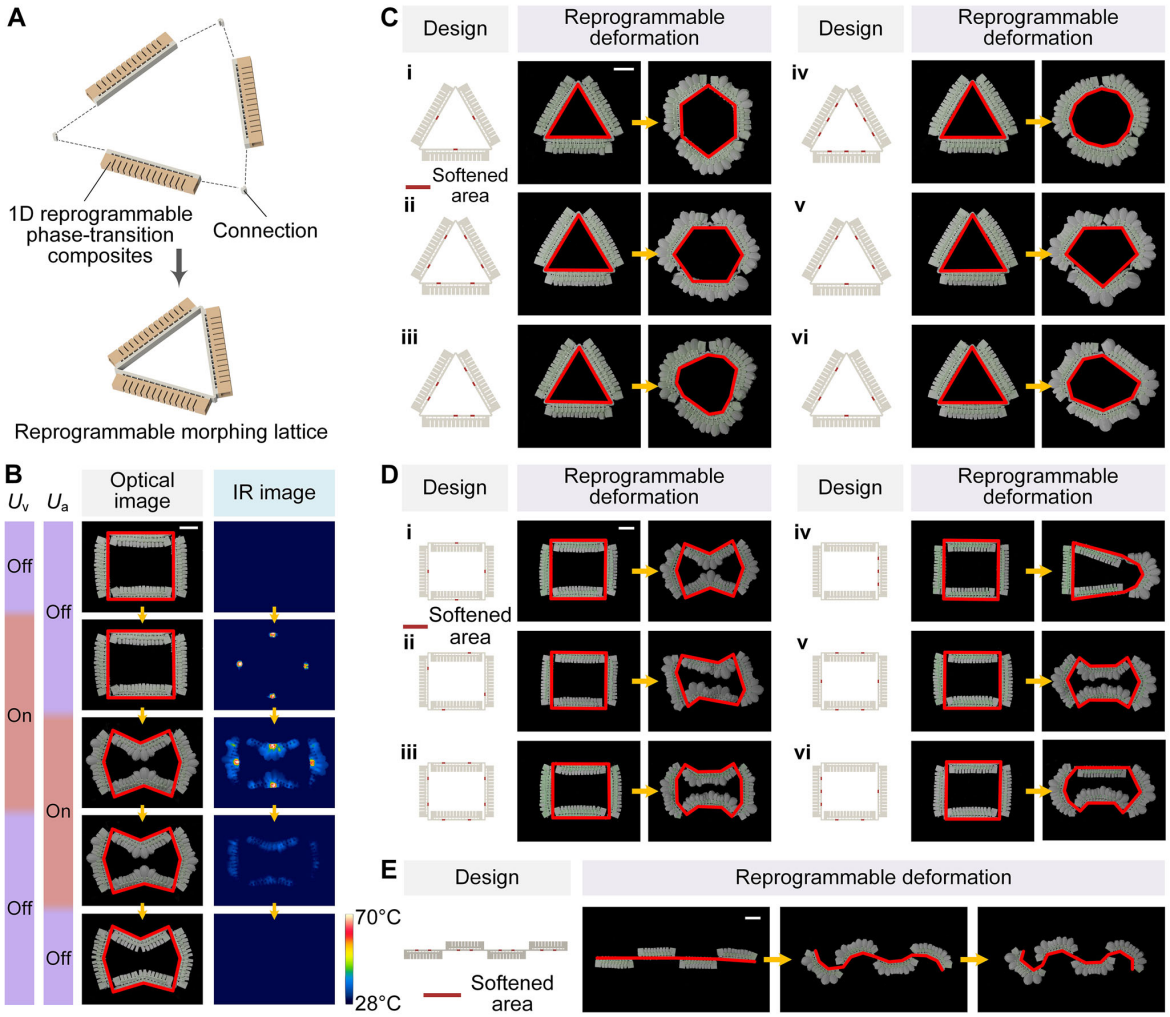
Reprogrammable morphing lattice. (A) Schematic structural composition of a reprogrammable morphing lattice composed of multiple 1D phase‐transition composites according to a patterned custom design. (B) Optical and IR image sequences of the shape locking process of a reprogrammable morphing lattice. *U*_v_ refers to the voltage applied to the stiffness control heating wire, and *U*_a_ refers to the voltage applied to the actuation heating wire. Different programming design schemes and deformation experiments of (C) triangular and (D) quadrilateral reprogrammable morphing lattices. (E) Programming design scheme and deformation experiment of tandem structure. The red line indicates the controlled deformation contour of the reprogrammable morphing lattice. Scale bars, 5 cm.

By customizing the pattern composed of multiple 1D structural phase‐transition composites, 2D reprogrammable morphing lattices can be constructed to achieve a rich variety of lattice patterns and deformation types (Figure 4A). Here, the triangular and quadrilateral reprogrammable morphing lattices were first demonstrated, as shown in Figure 4C,D, Movies S4, and S5. By independently controlling the programming scheme of the variable stiffness layer of each side of the reprogrammable morphing lattice, and setting the different numbers and positions of the segments in the variable stiffness layer of each side to soften, the lattice can be changed into a variety of different shapes. For example, the triangular reprogrammable morphing lattice can be changed into pentagon, hexagon, heptagon, circle and other shapes as a whole, and each side of the lattice can have the same or different number and position of deformation segments, and the deformation direction of each side can also be designed to be the same or different. The above demonstration proves that the use of lattice structure can give phase‐transition composites more diverse and complex deformation forms. As shown in Figure 4E and Movie S6, phase‐transition composites can also achieve deformation of multiple segments with different types and directions by being connected in series in a one‐dimensional structure. The reprogrammable morphing lattice also has the ability to lock its shape. As shown in Figure 4B and Movie S7, after the lattice was programmed to deform, the variable stiffness layer was first cooled while maintaining the actuated deformation angle. After the variable stiffness layer recovered to a high stiffness state, the actuation layer was cooled, thereby locking the deformation of the lattice. At this time, even if the residual gas in the actuation layer was released, the reprogrammable deformable lattice could still remain in the set deformation state. In this way, the continuous energy consumption required to maintain deformation by the liquid‐vapor phase transition actuation method is avoided.

### 2.5 Reconfigurable Antenna and Reprogrammable Morphing Surface

**FIGURE 5:**
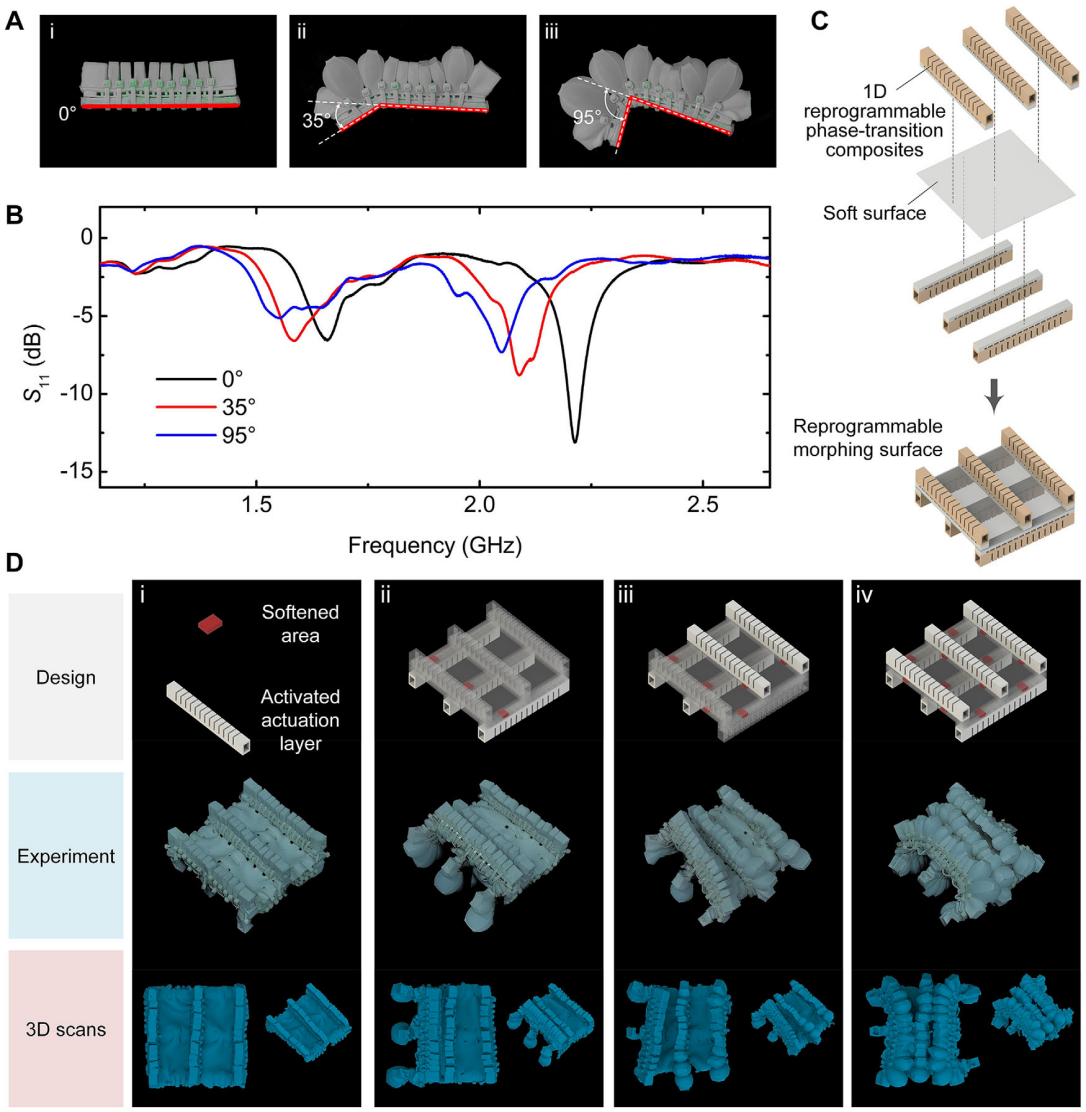
Reconfigurable antenna and reprogrammable morphing surface. (A) Images of the reconfigurable antenna in different deformation states. (B) Changes in the return loss *S*_11_ characteristics of the reconfigurable antenna at different bending angles, corresponding to the different states in (A). (C) Schematic diagram of the structural composition of the reprogrammable morphing surface. (D) Different programming designs and deformation experiments of reprogrammable morphing surface. The three rows of pictures show the programming design scheme, experimental deformation effect, and deformation 3D scanning effect, respectively.

Reconfigurable antennas [33, 34] can reconfigure their functions to meet the needs of different applications due to their ability to dynamically change their shape. Taking advantage of its reprogrammability and shape locking properties, we developed a reconfigurable antenna based on 1D phase‐transition composites. The structure of the reprogrammable antenna is similar to that of the 1D phase‐transition composites, but a section of copper wire is fixed between the heat insulation layer and the variable stiffness layer, which does not affect the normal morphing of phase‐transition composites. Moreover, as the shape of the phase‐transition composites changes, the shape of the fixed copper wire also changes accordingly, realizing the adjustment of the antenna performance. As shown in Figure 5A,B, taking 1‐point programming deformation as an example, the reconfigurable antenna achieved bending deformation at different angles, and the antenna's return loss *S*_11_ characteristic changed accordingly, proving that the antenna performance can be modulated by its shape morphing. In this way, we have achieved on‐demand programming deformation, shape locking, and corresponding performance tuning of the reconfigurable antenna using phase‐transition composites.

In addition to the aforementioned 1D and 2D phase‐transition composites, a reprogrammable morphing surface with a 3D structure that can achieve spatial deformation was developed based on phase‐transition composites. As shown in Figure 5C, the reprogrammable morphing surface was formed by arranging multiple 1D phase‐transition composites on both sides of the soft surface in sequence according to the design pattern. As shown in Figure 5D and Movie S8, by selectively activating part of the phase‐transition composites in the reprogrammable morphing surface and regulating the number and position of the softening section of its variable stiffness layer, the reprogrammable morphing surface can achieve rich and adjustable 3D spatial deformation. This further expands and proves the diverse reprogrammable deformation of phase‐transition composites.

### 2.6 Shape‐Shifting Amphibious Robot

**FIGURE 6:**
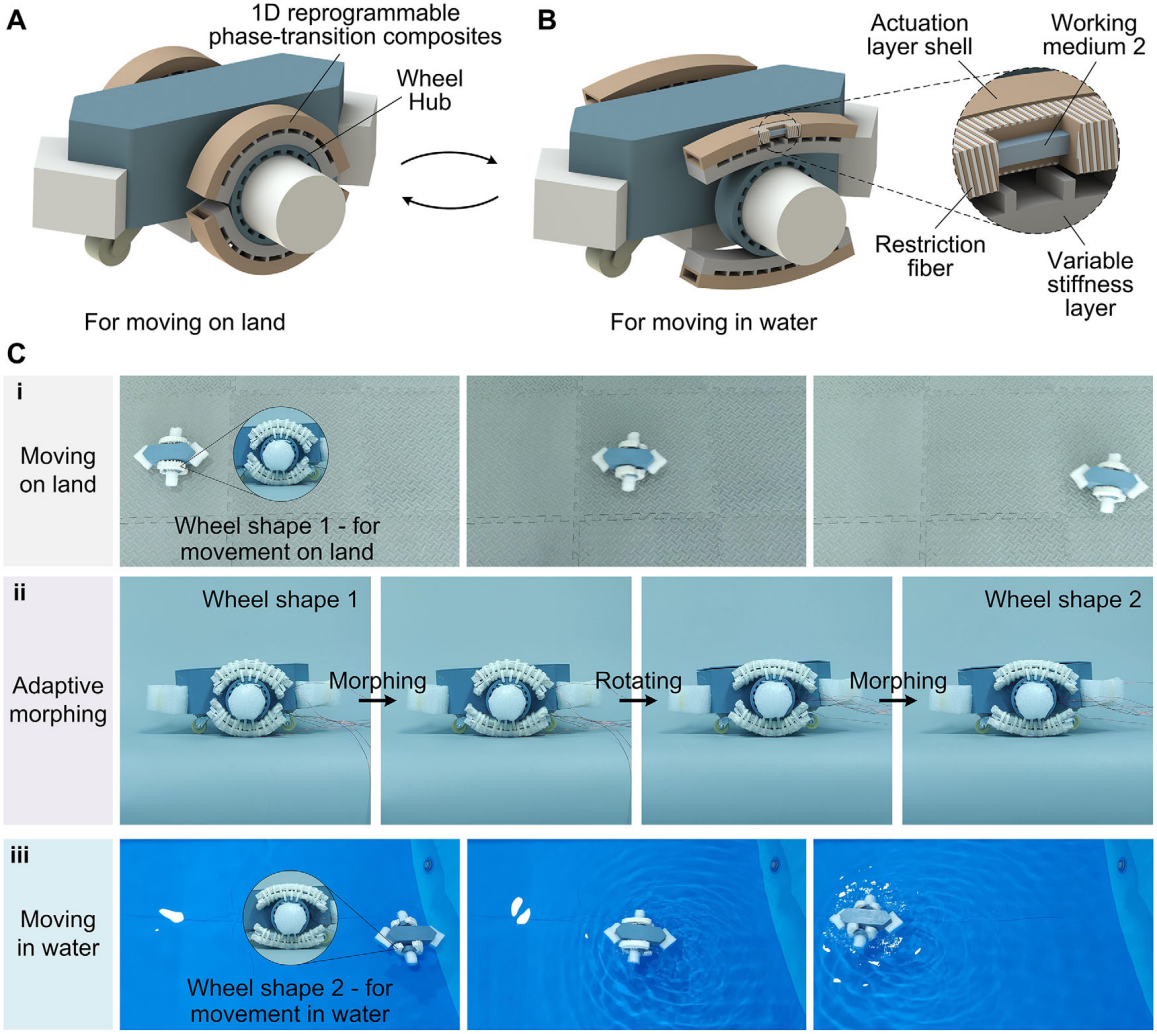
Shape‐shifting land‐water amphibious robot. Schematic diagram of (A) the state for movement on land and (B) the state for movement in water. The robot switched between these two states through adaptive deformation. (C) Demonstration of amphibious movement of the land‐water amphibious robot. The robot went through three states in sequence: (i) the robot's land motion under wheel shape 1; (ii) the adaptive morphing of the wheel shape; (iii) the robot's water motion under wheel shape 2.

Adaptive dynamic deformation is of great significance for robots to adapt to complex environments, and the development of reprogrammable phase‐transition composites provides a new idea for this. Using the reversible deformation and shape locking capabilities of phase‐transition composites, a land‐water amphibious robot that can reversibly adjust the shape of the wheels to adapt to different movement requirements on land and in water was first developed. As shown in Figure 6A,B and Figure S6, each deformable wheel was composed of two 1D reprogrammable phase‐transition composites wrapped around the wheel hub. When the phase‐transition composites fit the wheel hub, the deformable wheel was circular as a whole to adapt to rolling motion on land. At this time, the robot's movement speed on land was 37.9 cm/s (Figure 6C‐i), but the paddling efficiency in this state was low and it was not suitable for movement in water. In order to adapt to movement in water, the variable stiffness layer on one side was first heated. With the elasticity of the silicone shell and the effect of gravity, the phase‐transition composites on this side became straight and could fix its shape after the variable stiffness layer cooled down. Then, the wheel was rotated 180 degrees as a whole and the above operation was repeated on the other phase‐transition composites. In this way, when the phase‐transition composites straightened and unfolded, the deformable wheels deformed into a state suitable for efficient paddling, in which the robot moved at a speed of 9.5 cm/s in the water (Figure 6C‐iii). This demonstrates that the robot's motion adaptability in different media can be modulated by its shape morphing. The deformable wheel can also be switched to the state for land movement again by actuating the phase‐transition composites to deform so as to fit the wheel hub and then lock the shape. Here, the actuation layer of the phase‐transition composites was composed of a silicone actuation layer shell embedded with restriction fibers, the contained working medium 2 and actuation heating wires. As shown in Figure S3A, the robot's movement is achieved by motors driving the wheels to rotate. Therefore, the robot's movement speed in water and on land is mainly controlled by adjusting the motor speed, which can be achieved by adjusting the driving voltage applied to the motor. As shown in Figure 6C and Movie S9, the land‐water amphibious robot with wheel shape 1 first moved on land, and then the deformable wheels were switched to wheel shape 2 using the aforementioned method, and then the land‐water amphibious robot in this state moved in water. The above demonstration proved the ability of phase‐transition composites to achieve adaptive shape switching through reprogrammable deformation and shape locking.

**FIGURE 7:**
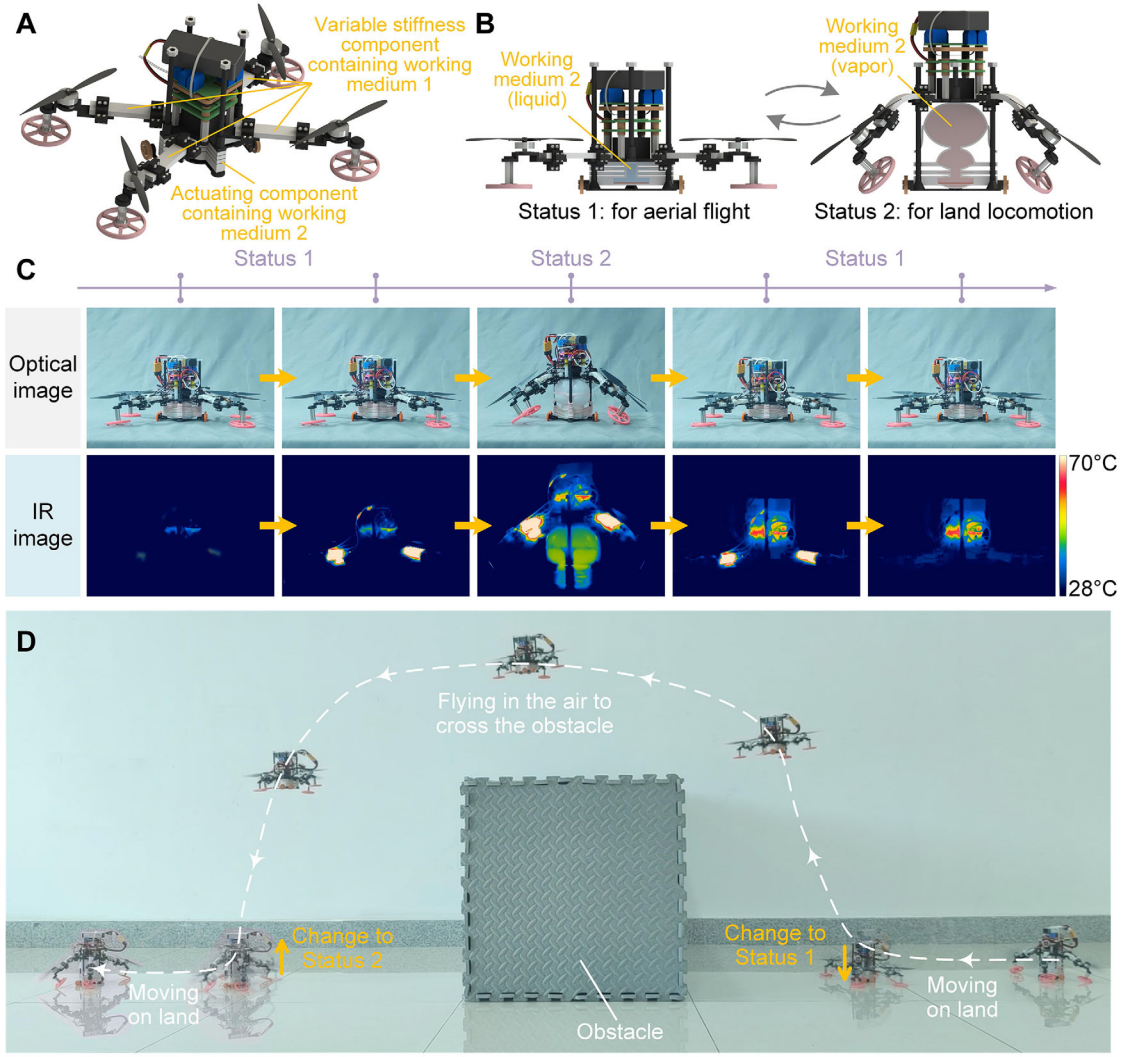
Shape‐shifting land‐air amphibious drone. (A) Schematic diagram of the structure of the land‐air amphibious drone. (B) Schematic diagram of the switching between the air flight state and the land locomotion state of the amphibious drone. (C) The process of the land‐air amphibious drone switching between two states through reversible deformation. (D) Demonstration of the land‐air amphibious drone crossing an obstacle by switching states. The drone sequentially achieved land locomotion—switching state—flying over an obstacle—switching state—continuing land locomotion.

In addition, we developed a land‐air amphibious drone. As shown in Figure 7A and Figure S7, the variable stiffness components containing working medium 1 were used as deformable arms, and the actuating component containing working medium 2 was used to drive the overall deformation of the drone structure. As shown in Figure 7B,C and Movie S10, after the set area of the variable stiffness components softened, the actuating component could drive the entire drone to reversibly lift/lower through the reversible liquid‐vapor phase transition of working medium 2, and the arms composed of the variable stiffness components underwent corresponding reversible deformation. In this way, the drone can reversibly switch between the state for aerial flight and the state for land locomotion, and can also be fixed in the switched state by restoring the variable stiffness component to a high stiffness state after the switching is completed. This amphibious capability is of great significance for the movement and work of drones in complex environments. Moving on land can reduce power consumption and thus extend the self‐sustaining time of drones, while flying in the air can have higher flexibility and can cross various obstacles. This demonstrates that the drone's energy consumption characteristics and obstacle‐crossing capabilities can be altered through its shape‐morphing‐based status switching. Combining these two states can make drones have both higher self‐sustaining ability and adaptability to complex environments. As shown in Figure S3B, the motion of the land‐air amphibious drone in Figure 7 is achieved by four motors driving their respective propellers. In status 1, used for air flight, each of the four propellers provides a downward force perpendicular to the ground as they rotate. By controlling the four propellers to provide different force outputs, controlled air flight can be achieved. In status 2, used for land locomotion, the component of the force perpendicular to the ground generated by the rotation of the four propellers is canceled out by the drone's gravity, while a component force parallel to the ground is provided for planar movement on land. By controlling the four propellers to provide different force outputs, the passive wheels are driven to roll on the ground, thus achieving controlled land movement of the drone. As a demonstration of this capability, as shown in Figure 7D, the land‐air amphibious drone first moved on land in state 2, then switched to state 1 to fly in the air to cross the obstacle, and then switched to state 2 again after landing, so as to continue moving forward on land. This also proves that the adaptive dynamic deformation of phase‐transition composites can endow robots with the ability to adapt to complex environments, and is of great significance for the future application of multifunctional reconfigurable robot systems.

## 3 Discussion

Adaptive dynamic deformation is of great significance for robots to adapt to complex environments, and flexible smart materials with rich deformation forms and the ability to accurately program and regulate deformation are an important way to achieve it. The method of adjusting the motion mode by passive deformation can only be reconfigured by external forces, and cannot reversibly and actively regulate the deformation. The active deformable robots in existing research are difficult to program and control, have few degrees of freedom in deformation, and can only deform uniformly as a whole. Designing a flexible smart material capable of reprogrammable and local programmable regulation for adaptive dynamic deformation in robotic systems is a substantial challenge. In this paper, inspired by the biological behavior of regulating growth shape through stiffness changes and providing growth momentum through fluid pressure, we proposed a reprogrammable phase‐transition composites that uses the stiffness change induced by reversible solid‐liquid phase transition to program and regulate the material deformation actuated by reversible liquid‐vapor phase transition. In this way, it can achieve adaptive dynamic deformation in a controllable manner, realize rich and diverse reprogrammable deformation and local programmed deformation, and perform adaptive deformation according to the target contour. By regulating the energy storage‐release process, the deformation response speed of the liquid‐vapor phase transition actuation method is greatly accelerated; by locking the deformation through the reversible solid‐liquid phase transition, the continuous energy consumption required for the liquid‐vapor phase transition actuation method to maintain the deformation is avoided. A series of functional enhancements and application demonstrations have proved the effectiveness of reprogrammable phase‐transition composites and their important application value for adaptive dynamic deformation of robotic systems. In phase‐transition composites, the outer shells that are in contact with the external environment are made of common silicone rubber, a material frequently used in human‐machine interface applications, ensuring the good safety and compatibility of our phase‐transition composites. Using working medium 2 with a lower boiling point can obtain a higher actuating pressure at the same temperature, but due to its higher saturated vapor pressure, its evaporation rate at room temperature is also faster. Future research will consider methods such as modifying the shell material or applying a hydrophobic coating to prevent leakage of the working medium 2, thereby further improving the long‐term stability of the phase‐transition composites. Compared with the existing flexible smart materials for deformable robots or structures [5, 17, 18, 19, 20, 27, 28, 29, 30] (Table S1), our phase‐transition composites have obvious advantages, including reversible active deformation control, reprogrammable deformation, local programmable deformation, shape locking, and rapid deformation. And compared with other actuation methods used in existing flexible smart materials for deformable robots, especially the most commonly used traditional flexible fluid (pneumatic/hydraulic) actuation method [35, 36, 37, 38, 39, 40], the phase‐transition actuation method [41, 42, 43] used here has high pressure and large deformation output while eliminating the external complex pump and valve system, which is conducive to its application in untethered robots. Our work is expected to provide a reference and new research direction for the research of deformable robot technology and phase‐transition actuation technology.

## 4 Methods

### 4.1 Materials and Fabrication of Reprogrammable Phase‐Transition Composites

As shown in Figure S1, the phase‐transition composites consist of an actuation layer, a heat insulation layer, and a variable stiffness layer. The actuation layer includes an actuation layer shell, a strain limiting layer, actuation heating wires, and working medium 2; the variable stiffness layer includes a variable stiffness layer shell, a stiffness control heating wire, and working medium 1; the heat insulation layer has a column array structural design to increase thermal resistance and avoid mutual thermal interference between the above two layers. The manufacturing of the phase change composite material included the following steps. (i) Fabrication of actuation layer: As shown in Figure S2A, first, silicone 5A (PS6600, Shenzhen Yipin Trading Co. Ltd., China) was poured into the mold and cured to obtain the actuation layer shell, and then the shell was bonded and assembled with the strain limiting layer and the actuation heating wire (constantan wire) according to the design, and finally the working medium 2 was injected to obtain the actuation layer. (ii) Fabrication of heat insulation layer: As shown in Figure S2B, the heat insulation layer was obtained by pouring silicone 30A (PS6600, Shenzhen Yipin Trading Co. Ltd., China) into the mold and curing it. (iii) Fabrication of variable stiffness layer: As shown in Figure S2C, the prefabricated working medium 1 core was first embedded in the variable stiffness layer shell using step‐by‐step pouring of silicone 30A. After demolding, the shell was pre‐stretched and the excess shell at both ends was cut off and the two ends were re‐sealed with Sil‐Poxy (Smooth‐On, USA) to obtain a semi‐finished product 2. Then, the working medium 1 core was heated and melted to release the pre‐strain of the shell. After the working medium 1 cooled and re‐solidified, the stiffness control heating wire was wound on the shell according to the design so that it could be independently controlled in sections. Then, silicone rubber was applied to the shell to fix the stiffness control heating wire, so that the variable stiffness layer was obtained. In this step, the working medium 1 core was obtained by casting in a prefabricated silicone soft mold after melting, and the pre‐stretching of the variable stiffness layer shell was to apply prestress to the working medium 1 to promote its healing in the liquid phase state. (iv) Assembly: Finally, as shown in Figure S2D, the phase‐transition composites can be manufactured by assembling the actuation layer, the heat insulation layer and the variable stiffness layer in sequence. The phase‐transition composites utilize the liquid‐vapor phase transition process of the working medium 2 and the solid‐liquid phase transition process of the working medium 1. Therefore, the boiling point of the working medium 2 and the melting point of the working medium 1 can be selected as needed to adjust the actuation temperature and stiffness control temperature of the phase‐transition composites. In the experiment, working medium 2 used fluids with relatively low boiling points, whose liquid‐vapor phase transition can produce high pressure (over 200 kPa [26]). The fluids we used includes Novec 7000 (boiling point 34°C), Fluere 747 (boiling point 47°C), ethanol (boiling point 78°C), and water (boiling point 100°C). Specifically, in Figures 4 and 5, ethanol was used as the working medium 2 for the reprogrammable morphing lattice, reconfigurable antenna, and reprogrammable morphing surface. In Figures 6 and 7, Fluere 747 was used as the working medium 2 for the shape‐shifting land‐water amphibious robot and shape‐shifting land‐air amphibious drone. Working medium 1 used alloys with relatively low melting points, whose stiffness can change significantly (over 1000 times [25]). Here we used an alloy with a melting point of 47°C (44.7%Bi, 22.6% Pb, 19.1%In, 8.3%Sn, 5.3%Cd). The reconfigurable antenna (Figure 5A,B) was fabricated by fixing a section of copper wire (20 cm long and 0.41 mm diameter) between the heat insulation layer and the variable stiffness layer. As shown in Figure S3A, the shell, frame, motor, circuit, battery, passive wheel, foam, whell hub and phase‐transition composites were assembled to obtain a land‐water amphibious robot. Here, the actuation layer shell of the phase‐transition composites adopted a configuration of embedded restriction fibers. As shown in Figure S3B, the frames, circuit, MOS, battery, passive wheels, motors, limit nuts, actuation component and variable stiffness component were assembled to manufacture a land‐water amphibious drone.

### 4.2 Characterization

An IR camera (A615, FLIR, USA) and a digital camera were used to obtain IR and optical information of the phase‐transition composites and its applications, respectively. The time response performance of the deformation angle of the phase‐transition composites in Figure 2 was obtained by analyzing the pictures taken by the digital camera using ImageJ. A vector network analyzer (LiteVNA) was used to measure the return loss *S*_11_ characteristics of the reconfigurable antenna (Figure 5B). The 3D shape scan of the reprogrammable morphing surface (Figure 5D) was performed using a 3D scanner (Creality 3D Technology Co., Ltd., China), and then the scanned information was processed using Geomagic Studio.

### 4.3 Simulation of Deformation

We used ABAQUS to perform finite element simulations to predict the deformation of phase‐transition composites under different conditions, thereby guiding their design. The pressure provided by the liquid‐vapor phase transition of the working medium 2 was applied to the actuation layer in an equivalent manner, that was, in ABAQUS, the pressure was applied as a load to the inner surface of the actuation layer shell. As its temperature rises, the working medium 2 undergoes a liquid‐vapor phase transition, and the increased internal pressure Δ*P* of the actuation layer can be described by the saturated vapor pressure *P_v_* (*T*) of the working medium 2:

(2) $$\Delta P=P_{v}\left(T_{0}+\Delta T\right)-P_{v}\left(T_{0}\right)$$

For a single‐component working medium 2, its saturated vapor pressure can be calculated using the Antoine equation [44]:

(3) $$\mathrm{lg}P_{v}=A-\frac{B}{T+C}$$

In the above formula, *A*, *B*, and *C* were coefficients to be determined by the type of working medium 2. For a multi‐component mixture of working medium 2, the saturated vapor pressure can be calculated according to Raoult's law:

(4) $$P_{v}=\frac{\sum_{i=1}^{N}n_{i}{P_{v}}_{-i}}{\sum_{i=1}^{N}n_{i}}$$

where *n_i_* was the amount of substance of the *i*‐th component, *P_v‐i_* was the saturated vapor pressure of the *i*‐th component, and *N* was the total number of components. In addition, the nonlinear elastic material properties of the silicone material involved in the phase‐transition composites were described by the hyperelastic incompressible Yeoh material model [45].

## Author Contributions

W.T. proposed and supervised the project. Y.Z. and W.T. designed the research. Y.Z., X.G., K.Q., P.Z., Q.S., and Y.W. conducted the experimental work. Y.Z. developed theoretical analysis, finite element simulation, and analyzed the data. Y.Z. and W.T. wrote the manuscript. W.T., J.Z., and H.Y. revised the manuscript.

## Conflicts of Interest

The authors declare no conflicts of interest.

## Supporting information




**Supporting File 1**: advs73901‐sup‐0001‐MovieS1.mp4.

**Supporting File 2**: advs73901‐sup‐0002‐MovieS2.mp4.

**Supporting File 3**: advs73901‐sup‐0003‐MovieS3.mp4.

**Supporting File 4**: advs73901‐sup‐0004‐MovieS4.mp4.

**Supporting File 5**: advs73901‐sup‐0005‐MovieS5.mp4.

**Supporting File 6**: advs73901‐sup‐0006‐MovieS6.mp4.

**Supporting File 7**: advs73901‐sup‐0007‐MovieS7.mp4.

**Supporting File 8**: advs73901‐sup‐0008‐MovieS8.mp4.

**Supporting File 9**: advs73901‐sup‐0009‐MovieS9.mp4.

**Supporting File 10**: advs73901‐sup‐0010‐MovieS10.mp4.

**Supporting File 11**: advs73901‐sup‐0011‐SuppMat.pdf.

## Data Availability

The data that support the findings of this study are available in the supplementary material of this article.

## References

[1] D. S. Shah , J. P. Powers , L. G. Tilton , S. Kriegman , J. Bongard , and R. Kramer‐Bottiglio , “A soft robot that adapts to environments Through shape change,” Nature Machine Intelligence 3 (2021): 51–59, 10.1038/s42256-020-00263-1.

[2] R. Baines , F. Fish , J. Bongard , and R. Kramer‐Bottiglio , “Robots that evolve on demand,” Nature Reviews Materials 9 (2024): 822–835, 10.1038/s41578-024-00711-z.

[3] D. Shah , B. Yang , S. Kriegman , M. Levin , J. Bongard , and R. Kramer‐Bottiglio , “Shape Changing Robots: Bioinspiration, Simulation, and Physical Realization,” Advanced Materials 33 (2021): 2002882, 10.1002/adma.202002882. 32954582

[4] M. Polzin , Q. Guan , and J. Hughes , “Robotic locomotion through active and passive morphological adaptation in extreme outdoor environments,” Science Robotics 10 (2025): adp6419, 10.1126/scirobotics.adp6419. 40009657

[5] R. Baines , S. K. Patiballa , J. Booth , et al., “Multi‐environment robotic transitions Through adaptive morphogenesis,” Nature 610 (2022): 283–289, 10.1038/s41586-022-05188-w. 36224418

[6] J. Che , X. Yang , J. Peng , J. Li , Z. Liu , and M. Qi , “Arc‐heating actuated active‐morphing insect robots,” Nature Communications 16 (2025): 3014, 10.1038/s41467-025-58258-8. PMC11950236 40148351

[7] L. V. Nguyen , H. Kim , K. T. Nguyen , F. Alambeigi , and V. A. Ho , “Adaptable cavity exploration: Bioinspired vibration‐propelled PufferFace Robot with a morphable body,” Science Advances 11 (2025): ads3006, 10.1126/sciadv.ads3006. PMC12042880 40305621

[8] X. Guo , W. Tang , K. Qin , et al., “Powerful UAV manipulation via bioinspired self‐adaptive soft self‐contained gripper,” Science Advances 10 (2024): adn6642, 10.1126/sciadv.adn6642. PMC11078182 38718123

[9] D. Wang , H. Hu , S. Li , et al., “Sensing‐triggered stiffness‐tunable smart adhesives,” Science Advances 9 (2023): adf4051, 10.1126/sciadv.adf4051. PMC10017039 36921055

[10] S. I. Rich , R. J. Wood , and C. Majidi , “Untethered soft robotics,” Nature Electronics 1 (2018): 102–112, 10.1038/s41928-018-0024-1.

[11] W. Tang , Y. Zhong , H. Xu , et al., “Self‐protection soft fluidic robots With rapid large‐area self‐healing capabilities,” Nature Communications 14 (2023): 6430, 10.1038/s41467-023-42214-5. PMC10576050 37833280

[12] W. Tang , C. Zhang , Y. Zhong , et al., “Customizing a self‐healing soft pump for robot,” Nature Communications 12 (2021): 2247, 10.1038/s41467-021-22391-x. PMC8046788 33854071

[13] Y. Zhong , W. Tang , H. Gui , et al., “Human camouflage and expression via soft mask from reprogrammable chemical fluid skin,” Science Advances 2025, 11, adq6141, 10.1126/sciadv.adq6141. PMC11818024 39937920

[14] Y. Jiang , S. Ji , J. Sun , et al., “A universal interface for plug‐and‐play assembly of stretchable devices,” Nature 614 (2023): 456–462, 10.1038/s41586-022-05579-z. 36792740

[15] Y. Li , N. Li , W. Liu , et al., “Achieving tissue‐level softness on stretchable electronics Through a generalizable soft interlayer design,” Nature Communications 14 (2023): 4488, 10.1038/s41467-023-40191-3. PMC10372055 37495580

[16] X. Cheng , Z. Fan , S. Yao , et al., “Programming 3D curved mesosurfaces using microlattice designs,” Science 379 (2023): 1225–1232, 10.1126/science.adf3824. 36952411

[17] D. Hwang , J. Barron Edward , A. B. M. T. Haque , and D. B. Michael , “Shape morphing mechanical metamaterials through reversible plasticity,”Science Robotics 7 (2022): abg2171, 10.1126/scirobotics.abg2171. 35138882

[18] E. Brown , N. Rodenberg , J. Amend , et al., “Universal robotic gripper based on the jamming of granular material,” Proceedings of the National Academy of Sciences 107 (2010): 18809–18814, 10.1073/pnas.1003250107.

[19] D.‐Y. Lee , J.‐K. Kim , C.‐Y. Sohn , J.‐M. Heo , and K.‐J. Cho , “High–load capacity origami transformable wheel,” Science Robotics 6 (2021): abe0201, 10.1126/scirobotics.abe0201. 34043563

[20] D. S. Shah , M. C. Yuen , L. G. Tilton , E. J. Yang , and R. Kramer‐Bottiglio , “Morphing Robots Using Robotic Skins That Sculpt Clay,” IEEE Robotics and Automation Letters 4 (2019): 2204–2211.

[21] P.‐F. Lenne and V. Trivedi , “Sculpting tissues by phase transitions,” Nature Communications 13 (2022): 664, 10.1038/s41467-022-28151-9. PMC8814027 35115507

[22] L. Beauzamy , N. Nakayama , and A. Boudaoud , “Flowers Under pressure: Ins and outs of turgor regulation in development,” Annals of Botany 114 (2014): 1517–1533, 10.1093/aob/mcu187. 25288632 PMC4204789

[23] A. Mongera , P. Rowghanian , H. J. Gustafson , et al., “A fluid‐to‐solid jamming transition underlies vertebrate body axis elongation,” Nature 561 (2018): 401–405, 10.1038/s41586-018-0479-2. 30185907 PMC6148385

[24] S. Kim , M. Pochitaloff , G. A. Stooke‐Vaughan , and O. Campàs , “Embryonic tissues as active foams,” Nature Physics 17 (2021): 859–866, 10.1038/s41567-021-01215-1. 34367313 PMC8336761

[25] Y. Hao , J. Gao , Y. Lv , and J. Liu , “Low Melting Point Alloys Enabled Stiffness Tunable Advanced Materials,” Advanced Functional Materials 32 (2022): 2201942, 10.1002/adfm.202201942.

[26] D. Fonseca and P. Neto , “Electrically‐driven phase transition actuators to power soft robot designs,” Nature Communications 16 (2025): 3920, 10.1038/s41467-025-59023-7. PMC12032282 40280926

[27] J. Sun , E. Lerner , B. Tighe , C. Middlemist , and J. Zhao , “Embedded shape morphing for morphologically adaptive robots,” Nature Communications 14 (2023): 6023, 10.1038/s41467-023-41708-6. PMC10533550 37758737

[28] L. Buckner Trevor , R. A. Bilodeau , Y. Kim Sang , and R. Kramer‐Bottiglio , “Roboticizing fabric by integrating functional fibers,” Proceedings of the National Academy of Sciences 117 (2020): 25360, 10.1073/pnas.2006211117. PMC7568323 32989123

[29] V. Maurin , Y. Chang , Q. Ze , S. Leanza , J. Wang , and R. R. Zhao , “Liquid Crystal Elastomer–Liquid Metal Composite: Ultrafast, Untethered, and Programmable Actuation by Induction Heating,” Advanced Materials 36 (2024): 2302765, 10.1002/adma.202302765. 37656872

[30] B. Yang , R. Baines , D. Shah , et al., “Reprogrammable soft actuation and shape‐shifting via tensile jamming,” Science Advances 7 (2021): abh2073, 10.1126/sciadv.abh2073. PMC11093226 34597130

[31] A. Nojoomi , J. Jeon , and K. Yum , “2D material programming for 3D shaping,” Nature Communications 12 (2021): 603, 10.1038/s41467-021-20934-w. PMC7841157 33504805

[32] W. Ai , J. Wu , Y. Long , and K. Song , “A Rolling Light‐Driven Pneumatic Soft Actuator Based on Liquid–Gas Phase Change,” Advanced Materials 37 (2025): 2418218, 10.1002/adma.202418218. 39924788

[33] J. S. Gibson , X. Liu , S. V. Georgakopoulos , J. J. Wie , T. H. Ware , and T. J. White , “Reconfigurable Antennas Based on Self‐Morphing Liquid Crystalline Elastomers,” IEEE Access 4 (2016): 2340–2348, 10.1109/ACCESS.2016.2565199.

[34] J. M. Kovitz , H. Rajagopalan , and Y. Rahmat‐Samii , “Design and Implementation of Broadband MEMS RHCP/LHCP Reconfigurable Arrays Using Rotated E‐Shaped Patch Elements,” IEEE Transactions on Antennas and Propagation 63 (2015): 2497–2507, 10.1109/TAP.2015.2417892.

[35] T. J. Jones , E. Jambon‐Puillet , J. Marthelot , and P. T. Brun , “Bubble casting soft robotics,” Nature 599 (2021): 229–233, 10.1038/s41586-021-04029-6. 34759362

[36] D. Melancon , B. Gorissen , C. J. García‐Mora , C. Hoberman , and K. Bertoldi , “Multistable inflatable origami structures at the metre scale,” Nature 592 (2021): 545–550, 10.1038/s41586-021-03407-4. 33883736

[37] J. Zou , M. Feng , N. Ding , et al., “Muscle‐fiber array inspired, multiple‐mode, pneumatic artificial muscles through planar design and one‐step rolling fabrication,” National Science Review 8 (2021): nwab048, 10.1093/nsr/nwab048. 34858608 PMC8566179

[38] Z. Zhang , Y. Long , G. Chen , Q. Wu , H. Wang , and H. Jiang , “Soft and lightweight fabric enables powerful and high‐range pneumatic actuation,” Science Advances 9 (2023): adg1203, 10.1126/sciadv.adg1203. PMC10096572 37043577

[39] H. Yuk , S. Lin , C. Ma , M. Takaffoli , N. X. Fang , and X. Zhao , “Hydraulic hydrogel actuators and robots optically and sonically camouflaged in water,” Nature Communications 8 (2017): 14230, 10.1038/ncomms14230. PMC5296644 28145412

[40] D. R. Higueras‐Ruiz , M. W. Shafer , and H. P. Feigenbaum , “Cavatappi artificial muscles From drawing, twisting, and coiling polymer tubes,” Science Robotics 6 (2021): abd5383, 10.1126/scirobotics.abd5383. 34043560

[41] Y. Zhong , W. Tang , C. Zhang , et al., “Programmable thermochromic soft actuators With “two dimensional” bilayer architectures for soft robotics,” Nano Energy 102 (2022): 107741, 10.1016/j.nanoen.2022.107741.

[42] Y. Zhong , W. Tang , H. Xu , et al., “Phase‐transforming mechanical metamaterials with dynamically controllable shape‐locking performance,” National Science Review 10 (2023): nwad192, 10.1093/nsr/nwad192. 37565196 PMC10411672

[43] W. Ai , K. Hou , J. Wu , Y. Long , and K. Song , “Miniaturized and untethered McKibben muscles based on photothermal‐induced gas‐liquid transformation,” Nature Communications 15 (2024): 1329, 10.1038/s41467-024-45540-4. PMC10864313 38351311

[44] G. W. Thomson , “The Antoine Equation for Vapor‐pressure Data,” Chemical Reviews 38 (1946): 1–39, 10.1021/cr60119a001. 21016992

[45] O. H. Yeoh , “Some Forms of the Strain Energy Function for Rubber,” Rubber Chemistry and Technology 66 (1993): 754, 10.5254/1.3538343.

