# Supplementary material for: Reprogrammable Phase‐Transition Composites for Adaptive Dynamic Shape Morphing
Source: Adv Sci (Weinh). 2026 Jan 21;13(17):e23219. doi: 10.1002/advs.202523219 (PMC13042661; doi:10.1002/advs.202523219)
Supplement: Supplementary file 11 — Supporting File 11: advs73901‐sup‐0011‐SuppMat.pdf. [file ADVS-13-e23219-s010.pdf]

Supplementary Materials

Reprogrammable phase-transition composites for adaptive dynamic shape morphing

Yiding Zhong et al.

*Corresponding author. Email: weitang@zju.edu.cn (Wei Tang);
junzou@zju.edu.cn (Jun Zou)

This PDF file includes:

Fig. S1. Schematic diagram of the layers of phase-transition composites.
Fig. S2. Fabrication process of phase-transition composites.
Fig. S3. Fabrication process of shape-shifting amphibious robot.
Fig. S4. Programmed deformation and shape-locking of phase-transition composites.
Fig. S5. Cyclic bending-recovery process of phase-transition composites in rapid deformation mode.
Fig. S6. Image of shape-shifting land-water amphibious robot.
Fig. S7. Image of shape-shifting land-air amphibious drone.
Table S1. Comparison of our phase-transition composites and existing flexible smart materials for deformable robots or structures.
Supplementary References

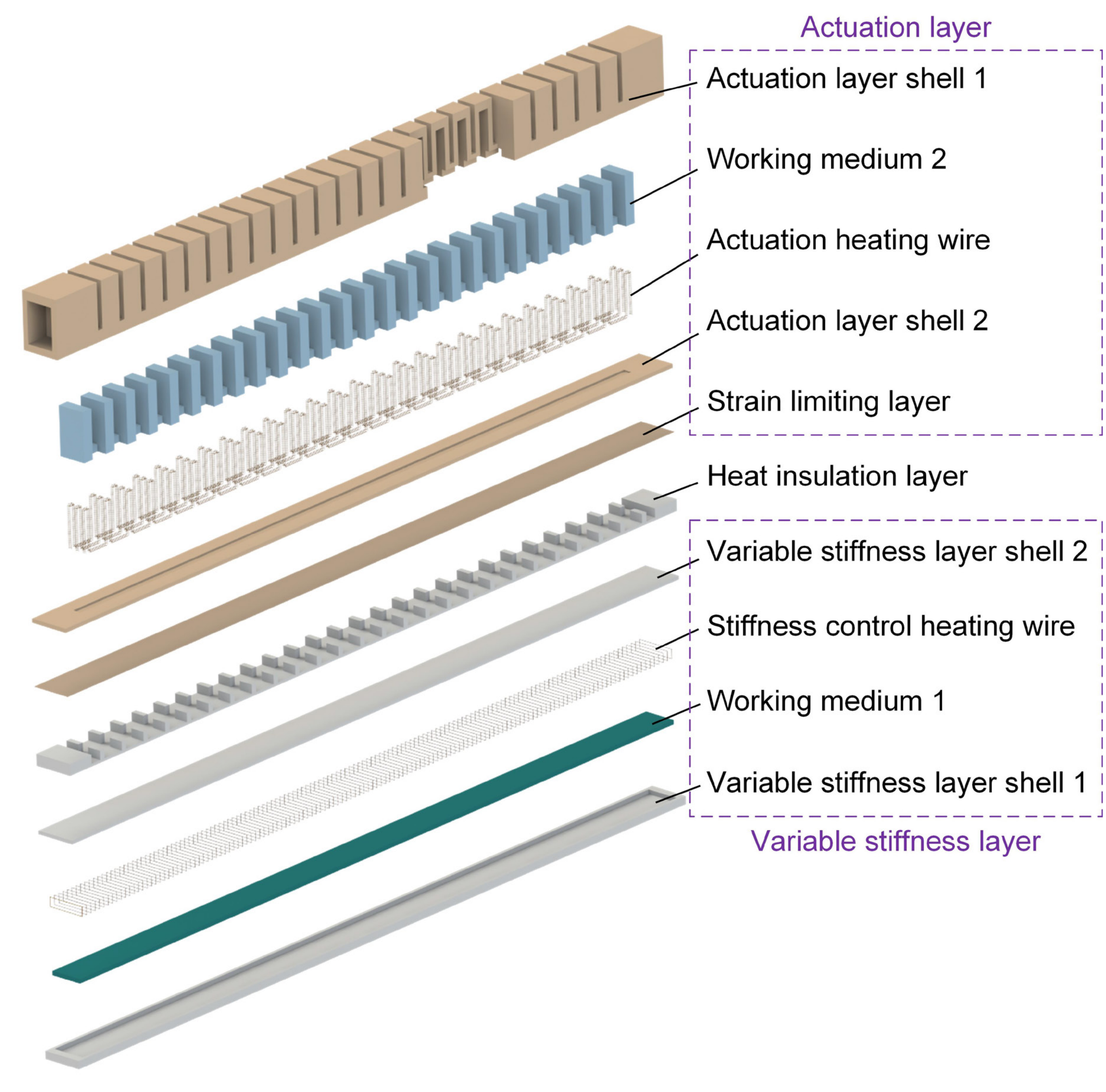

Fig. S1. Schematic diagram of the layers of phase-transition composites. The phase-transition composites mainly consist of 3 parts: (i) actuation layer, (ii) heat insulation layer, (iii) variable stiffness layer.

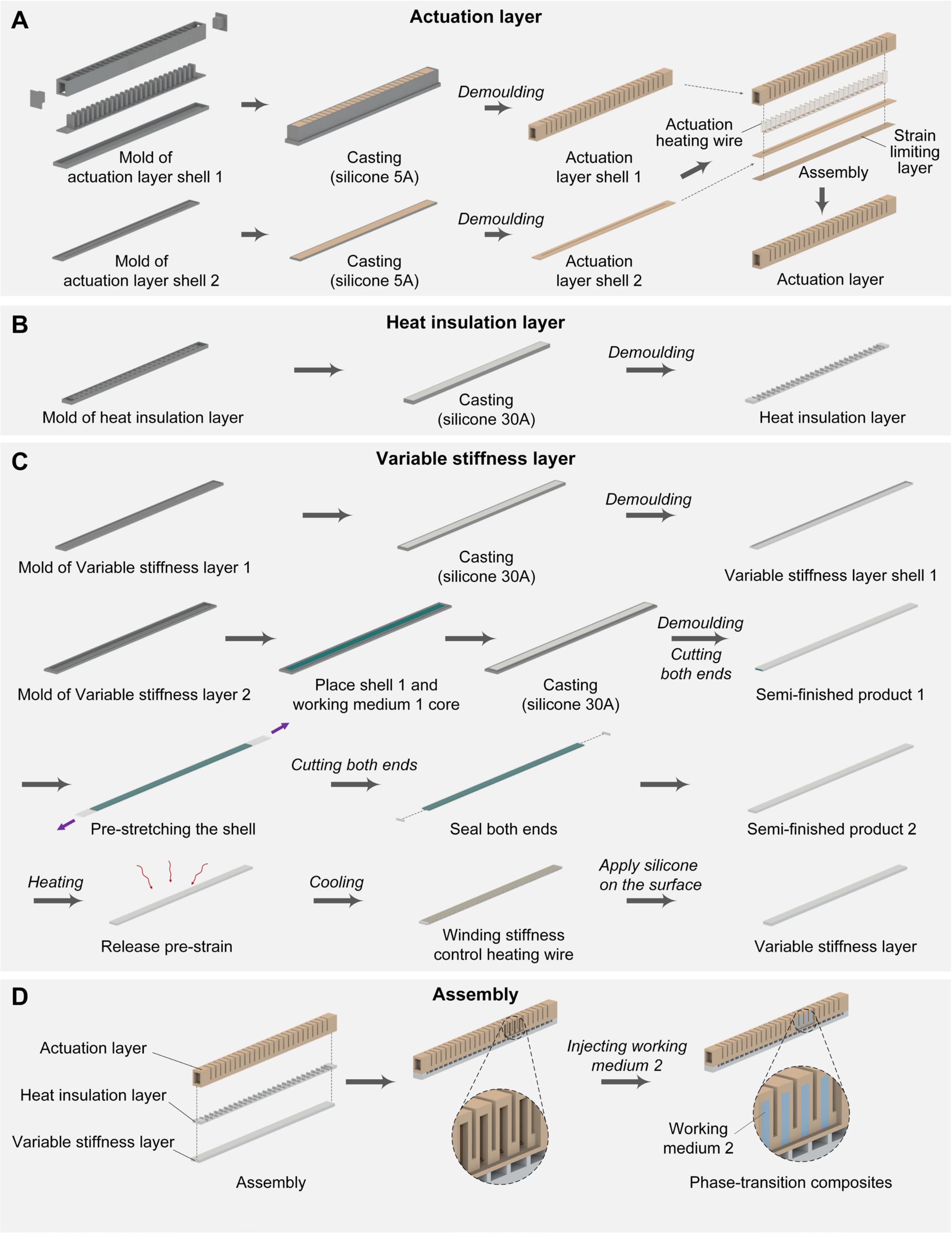

Fig. S2. Fabrication process of phase-transition composites. (**A**) Fabrication of actuation layer. (**B**) Fabrication of heat insulation layer. (**C**) Fabrication of variable stiffness layer. (**D**) Assembly.

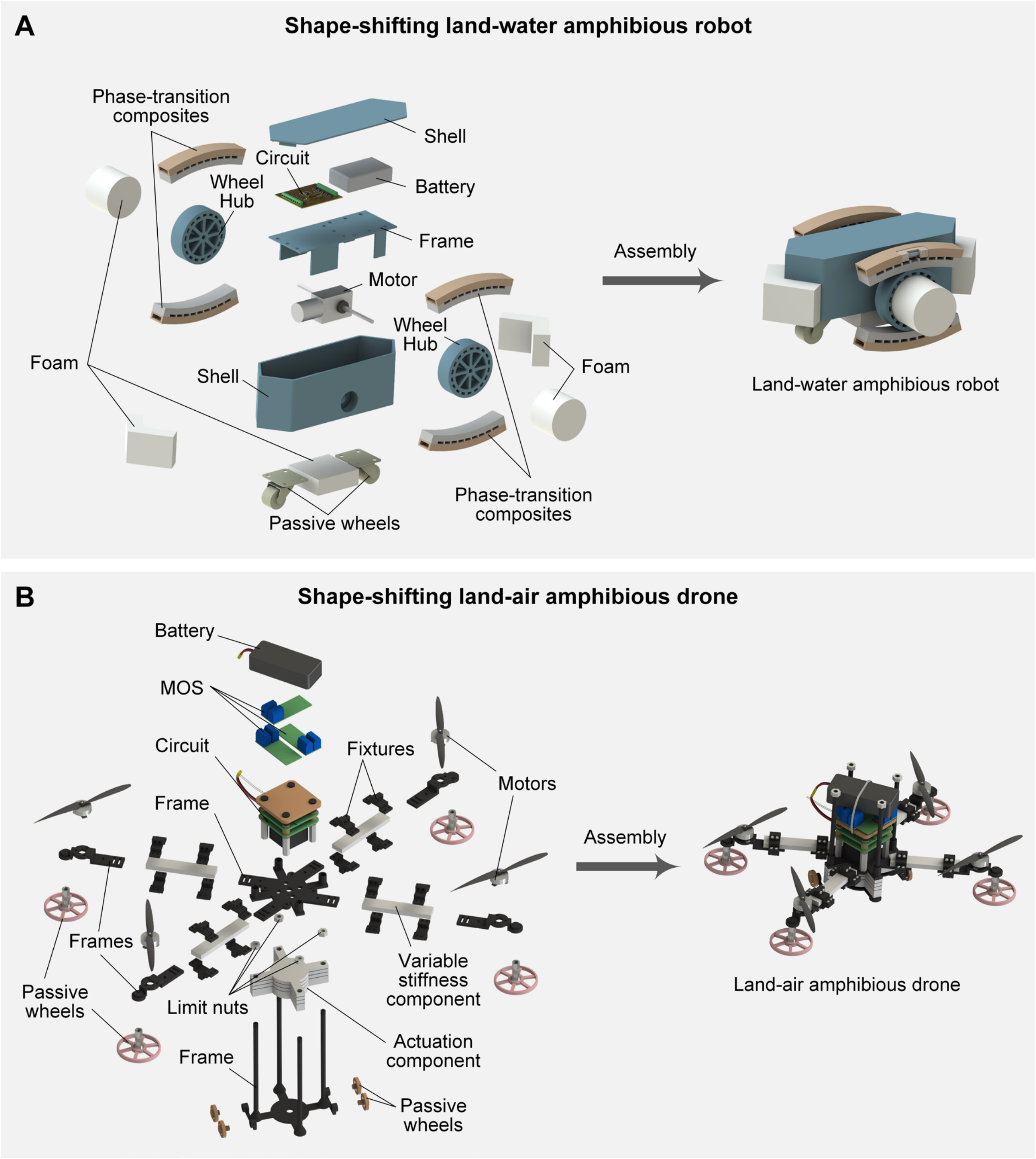

Fig. S3. Fabrication process of shape-shifting amphibious robot. (**A**) Fabrication of land-water amphibious robot. (**B**) Fabrication of land-air amphibious drone.

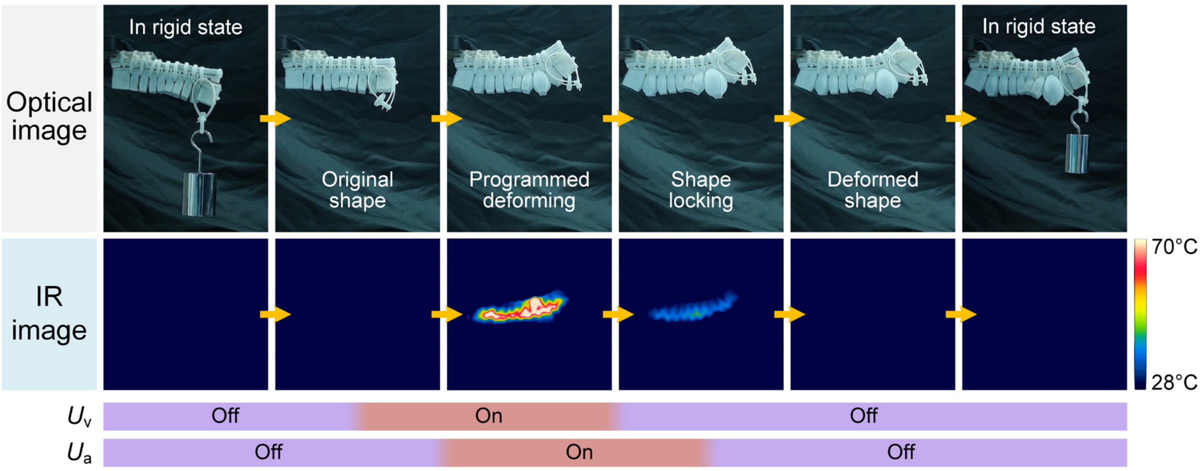

Fig. S4. Programmed deformation and shape-locking of phase-transition composites. By regulating the variable stiffness voltage (U_v) and the actuation voltage (U_a), the phase-transition composites could be programmed to change from the original shape to the deformed shape and lock the deformation. The phase-transition composites could withstand hanging weights in rigid states.

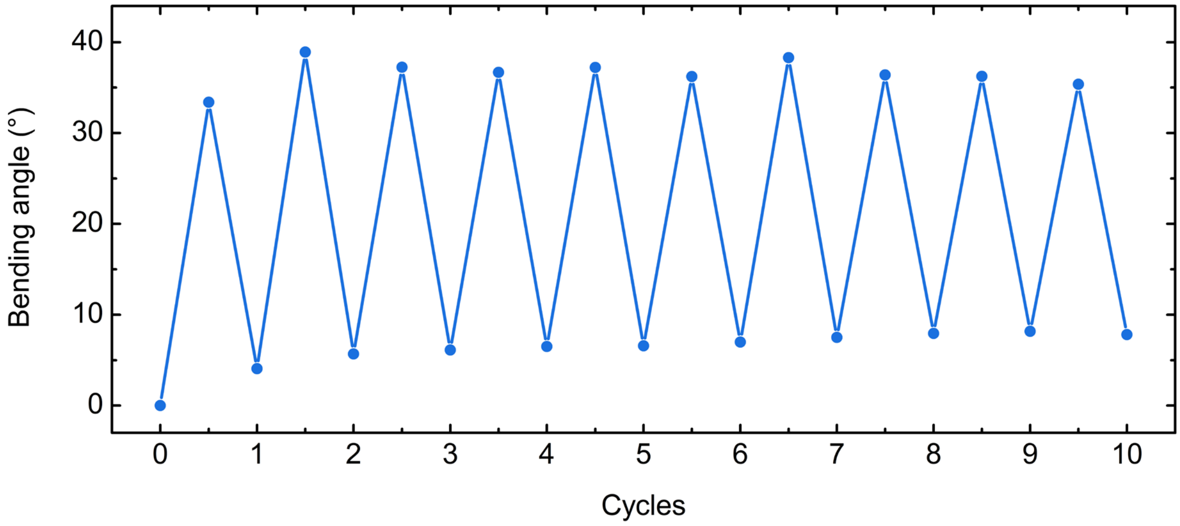

Fig. S5. Cyclic bending-recovery process of phase-transition composites in rapid deformation mode.

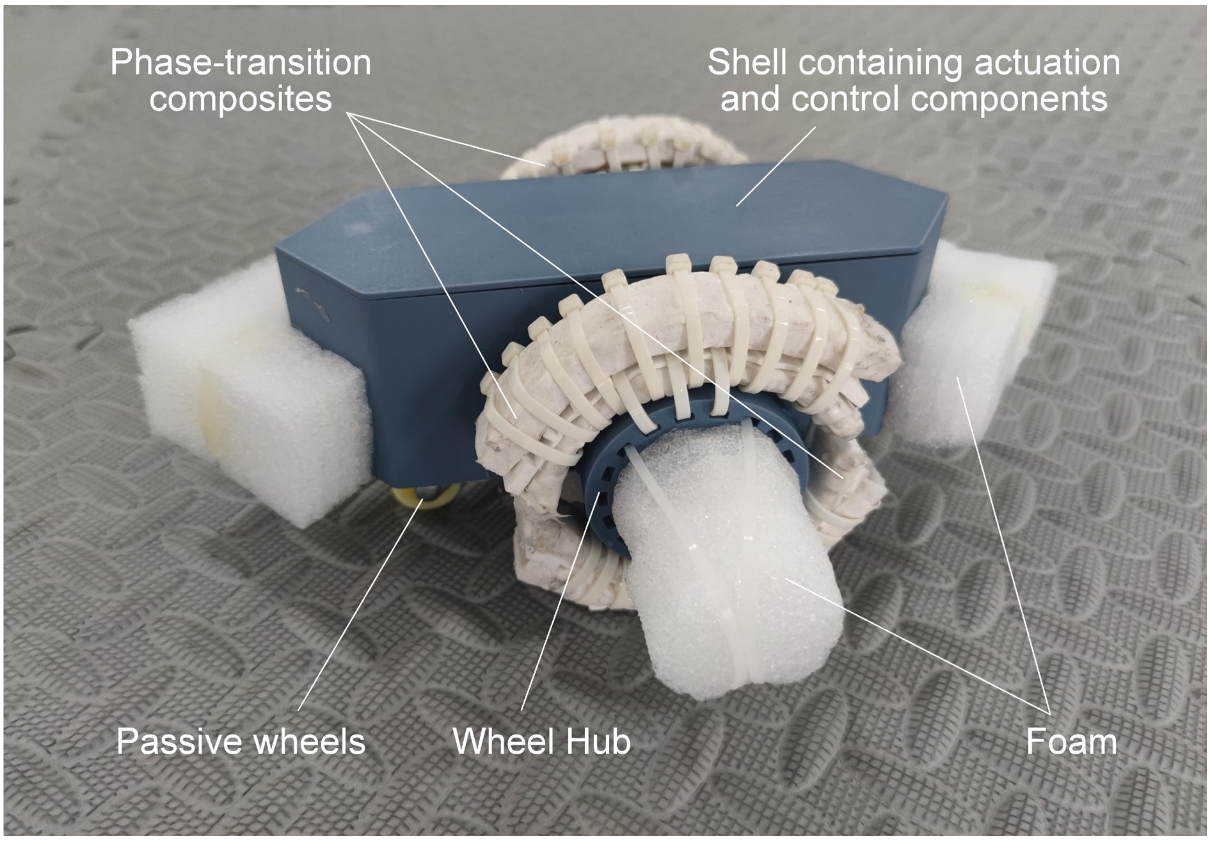

Fig. S6. Image of shape-shifting land-water amphibious robot. The land-water amphibious robot mainly consists of 3 parts: (i) deformable wheels composed of wheel hub and phase-transition composites, (ii) **s**hell containing actuation and control components, (iii) accessory components include foam and passive wheels.

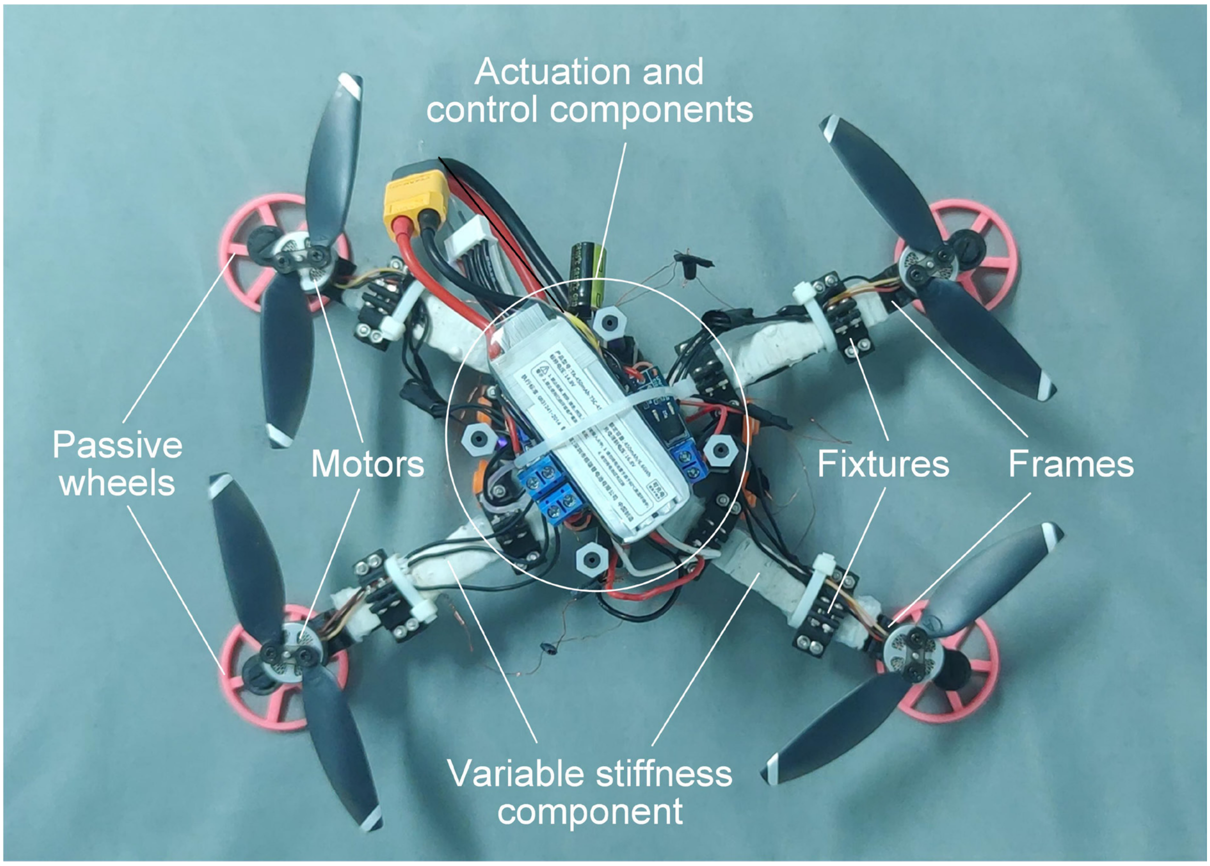

Fig. S7. Image of shape-shifting land-air amphibious drone. The land-air amphibious drone mainly consists of 3 parts: (i) **a**ctuation and control components include actuation component, frames, circuit, MOS and battery, (ii) deformable drone arms made of variable stiffness components, (iii) accessory components include passive wheels, motors, frames and fixtures.

Table S1. Comparison of our phase-transition composites and existing flexible smart materials for deformable robots or structures.

	**Reversible active deformation control**	**Reprogrammable deformation**	**Local programmable deformation**	**Shape locking**	**Rapid deformation**	**Untethered operation**	**Deformation actuation method**
Autonomously morphing drone[1]	×	×	×	✓	×	✓	/
Adaptive gripper[2]	×	×	×	×	×	×	Pneumatic actuator
Origami transformable wheel[3]	✓	×	×	✓	×	×	Hydraulic actuator
Clay sculpting robot skin[4]	✓	×	×	×	×	×	Pneumatic actuator
Turtle-inspired amphibious robot[5]	✓	×	×	✓	×	×	Pneumatic actuator
Morphologically adaptive robot[6]	✓	×	×	✓	×	✓	Twisted-and-coiled actuator
Roboticizing fabric[7]	✓	×	×	✓	×	✓	Shape memory alloy
Liquid crystal elastomer–liquid metal composite[8]	✓	×	✓	×	✓	✓	Liquid crystal elastomer
Jamming programming deformable surface[9]	✓	✓	×	×	×	×	Pneumatic actuator
**Phase-transition composites (This work)**	✓	✓	✓	✓	✓	✓	Reversible liquid-vapor phase transition

Note: "✓" means having this ability, while "×" means not having this ability.

Supplementary References

[1] D. Hwang, J. Barron Edward, A. B. M. T. Haque, D. Bartlett Michael, *Sci. Robot.* **2022**, *7*, eabg2171.

[2] E. Brown, N. Rodenberg, J. Amend, A. Mozeika, E. Steltz, M. R. Zakin, H. Lipson, H. M. Jaeger, *Proc. Natl. Acad. Sci. U.S.A.* **2010**, *107*, 18809.

[3] D.-Y. Lee, J.-K. Kim, C.-Y. Sohn, J.-M. Heo, K.-J. Cho, *Sci. Robot.* **2021**, *6*, eabe0201.

[4] D. S. Shah, M. C. Yuen, L. G. Tilton, E. J. Yang, R. Kramer-Bottiglio, *IEEE Robot. Autom. Lett.* **2019**, *4*, 2204.

[5] R. Baines, S. K. Patiballa, J. Booth, L. Ramirez, T. Sipple, A. Garcia, F. Fish, R. Kramer-Bottiglio, *Nature* **2022**, *610*, 283.

[6] J. Sun, E. Lerner, B. Tighe, C. Middlemist, J. Zhao, *Nat. Commun.* **2023**, *14*, 6023.

[7] L. Buckner Trevor, R. A. Bilodeau, Y. Kim Sang, R. Kramer-Bottiglio, *Proc. Natl. Acad. Sci. U.S.A.* **2020**, *117*, 25360.

[8] V. Maurin, Y. Chang, Q. Ze, S. Leanza, J. Wang, R. R. Zhao, *Adv. Mater.* **2024**, *36*, 2302765.

[9] B. Yang, R. Baines, D. Shah, S. Patiballa, E. Thomas, M. Venkadesan, R. Kramer-Bottiglio, *Sci. Adv.* **2021**, *7*, eabh2073.
